# Pyrethroids in the Home: Nondietary Pesticide Exposure in Children

**Published:** 2006-09

**Authors:** Julia R. Barrett

Because pyrethroid pesticides are often used in conventional agriculture, people are routinely exposed to trace amounts in foods. Similar exposure to organophosphorus (OP) pesticides has been described previously in results from the Children’s Pesticide Exposure Study, an investigation of pesticide exposures among 23 Seattle children aged 3–11. Unlike OP pesticides, however, pyrethroids are also approved for residential use. The latest findings from this study show that residential use of pyrethroids appears to be a more significant source of exposure to this class of pesticides than diet **[*EHP* 114:1419–1423; Lu et al.]**.

With the phaseout of residential use of the commonly used OP pesticides chlorpyrifos and diazinon, home use of pyrethroids has increased. Depending on the compound and the dose, pyrethroids may affect neurological development, disrupt hormones, induce cancer, and suppress the immune system. However, little is known about the extent and effects of human exposure.

Using samples collected during the summer of 2003, researchers at Emory University and the CDC determined urinary pyrethroid metabolite levels during 15 consecutive days for each child. During days 1–3 and 9–15, the children consumed foods prepared from conventionally grown crops. On days 4–8, organic items were substituted for plant-based foods such as fruits, vegetables, pasta, and cereal.

During the entire 15-day sampling period, the dominant metabolite seen was PBA, a nonspecific metabolite of permethrin, cypermethrin, and deltamethrin. PBA was detected in 82% of samples and had the highest median concentration, 0.45 μg/L. *trans*-DCCA and *cis*-DCCA, metabolites of permethrin, cypermethrin, and cyfluthrin, were also common, detected in 71% and 35% of all samples, respectively. Concentrations of *cis*-DCCA were too low to quantify; the median *trans*-DCCA concentration was 0.38 μg/L. The metabolites FPBA, derived from cyfluthrin, and DBCA, derived from deltamethrin, were each detected in only 2% of samples.

Comparing metabolites between dietary phases, the researchers saw no apparent trend. However, seven children in families that reported using pyrethroid pesticides had significantly higher levels of PBA and *trans*-DCCA than the other children and accounted for most of the FPBA-containing samples and all of the DBCA-containing samples. Interestingly, the older children experienced higher exposure than the younger ones. Typically younger children have higher exposure due to behaviors such as mouthing items and playing on floors, but the older children in this study spent time at sports facilities where pyrethroids may have been used.

The researchers conclude that an organic diet alone is unlikely to dramatically decrease a child’s exposure to pyrethroids the way it does exposure to OP pesticides. Limiting residential use of pyrethroids and preventing children’s contact with treated areas are very likely the best measures for decreasing their exposure to these pesticides.

## Figures and Tables

**Figure:**
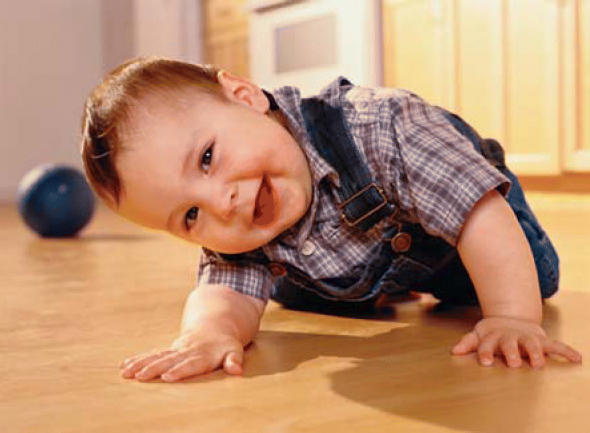
Close to the source New data show that OP pesticides used in the home contribute more to children’s exposure than diet.

